# Exploring the Mechanism of Inhibition of Au Nanoparticles on the Aggregation of Amyloid-β(16-22) Peptides at the Atom Level by All-Atom Molecular Dynamics

**DOI:** 10.3390/ijms19061815

**Published:** 2018-06-20

**Authors:** Menghua Song, Yunxiang Sun, Yin Luo, Yanyan Zhu, Yongsheng Liu, Huiyu Li

**Affiliations:** 1 College of Mathematics and Physics, Shanghai University of Electric Power, Shanghai 200090, China; songmenghua94@163.com (M.S.); yyzhu027@163.com (Y.Z.); ysliu@shiep.edu.cn (Y.L.); 2 College of Energy and Mechanical Engineering, Shanghai University of Electric Power, Shanghai 200090, China; 3 State Key Laboratory of Surface Physics, Key Laboratory of Computational Physical Sciences (Ministry of Education), and Department of Physics, Fudan University, 220 Handan Road, Shanghai 200433, China; wlxsunyunxiang@gmail.com (Y.S.); luoyin1986@gmail.com (Y.L.)

**Keywords:** Au nanoparticles, amyloid beta, peptide aggregation, inhibition mechanism, hydrophobic interaction, all-atom molecular dynamics simulations

## Abstract

The abnormal self-assembly of the amyloid-β peptide into toxic β-rich aggregates can cause Alzheimer’s disease. Recently, it has been shown that small gold nanoparticles (AuNPs) inhibit Aβ aggregation and fibrillation by slowing down the nucleation process in experimental studies. However, the effects of AuNPs on Aβ oligomeric structures are still unclear. In this study, we investigate the conformation of Aβ(16-22) tetramers/octamers in the absence and presence of AuNPs using extensive all-atom molecular-dynamics simulations in explicit solvent. Our studies demonstrate that the addition of AuNPs into Aβ(16-22) solution prevents β-sheet formation, and the inhibition depends on the concentration of Aβ(16-22) peptides. A detailed analysis of the Aβ(16-22)/Aβ(16-22)/water/AuNPs interactions reveals that AuNPs inhibit the β-sheet formation resulting from the same physical forces: hydrophobic interactions. Overall, our computational study provides evidence that AuNPs are likely to inhibit Aβ(16-22) and full-length Aβ fibrillation. Thus, this work provides theoretical insights into the development of inorganic nanoparticles as drug candidates for treatment of AD.

## 1. Introduction

Protein and peptide amyloid aggregation are related to more than 35 degenerative diseases, including Alzheimer’s (AD), Parkinson’s, Huntington’s and type 2 diabetes [1]. Among these diseases, AD is the most common neurodegenerative disorder with sensile plaques constituted by amyloid-β (Aβ) protein, with a length in residue ranging from 39–43, in patients’ brain tissues [2]. Aβ40 and Aβ42 are the predominant components of amyloid deposits in the brains of AD patients [2,3]. It has been generally recognized that Aβ aggregation from monomers toward amyloid fibrils largely follows a nucleation-growth mode [4]. Depending on the intrinsic misfolding property of Aβ and external environmental conditions, Aβ aggregation often produces many on-pathway and off-pathway species of different sizes, structures and functions via complex aggregation pathways. The aggregation of Aβ is a nucleation-elongation process with an “all-or-none” sigmoidal kinetics; a lag phase of the formation of a critical nucleus, followed by fibril elongation, proceeds rapidly via sequential additions of monomer [5]. During the nucleation phase, Aβ can form a variety of metastable, heterogeneous intermediate states [6]. The aggregation between the amyloidogenic core region displayed a critical role in the early conformational transition and the following oligomerization toward Aβ fibrillation. A prior study revealed that the central hydrophobic core (CHC) Aβ(17-20) (i.e., ^17^LVFF^20^) played a significant role in β-sheet formation of full-length Aβ [7]. An experimental study found the fibril formed by the Aβ(16-22) segment (i.e., ^16^KLVFFAE^22^) was similar to the Aβ full-length fibrils, for example the Aβ16-22 fibril could seed Aβ40/42 aggregation [8]. Thus, to investigate the mechanism of nanoparticle-mediated aggregation of Aβ peptides, Aβ(16-22) is an ideal model.

Numerous areas of science and technology have been significantly impacted by the fast-developing field of nanotechnology. Among these are previously-reported nanomaterials, such as fullerene [9,10], carbon nanotubes [11,12] and polymeric [13] and gold nanoparticles (AuNPs) [14,15,16]. Naturally, AuNPs have become one of the most outstanding candidates in different practical applications and fundamental research studies [15,17]. AuNPs have been extensively explored for biomedical applications due to their advantages of facile synthesis and surface functionalization [18,19]. Previous studies have suggested that AuNPs show a synergistic effect in inhibiting Aβ aggregation, dissociating Aβ fibrils and decreasing Aβ-mediated peroxidase activity and Aβ-induced cytotoxicity [20,21,22]. Gao et al. found that large AuNPs accelerate Aβ aggregation, whereas small AuNPs could significantly postpone or even completely inhibit this process [16]. However, the mechanism of how AuNPs regulate Aβ peptides aggregation is still elusive.

In this work, we performed extensive atomistic molecular dynamics (MD) simulations of the Aβ(16-22) tetramer and octamer in explicit solvent with and without AuNPs. The reason for choosing a tetramer and an octamer is that we want to see the different effects of AuNPs on different concentrations of Aβ(16-22) peptides, and the minimum nucleus size consists of at least eight Aβ(16-22) peptide chains based on the stability of the performed β-sheet assemblies [23]. Our aim is to characterize the structures of Aβ(16-22) peptides with different concentrations in the absence and presence of AuNPs, thereby providing theoretical insights into the development of drug candidates for AD.

## 2. Results and Discussion

To characterize the structures of the Aβ(16-22) tetramer, Aβ(16-22) octamer, Aβ(16-22) tetramer + AuNPs and Aβ(16-22) octamer + AuNPs, two MD runs, each of 500 ns, were carried out for each system. We discarded the first 100 ns of each simulation to remove the bias of the initial states, except when mentioned. In each system, the total simulation time was 500 ns. Therefore, the conformational properties were based on 3.2 μs.

The convergence of the four MD simulations was examined by comparing the following parameters within two different time intervals (300–350 ns and 350–400 ns) for all simulations. As shown in Figure S1, the number of H-bonds of the Aβ peptides within the two time intervals (300–350 ns and 350–400 ns) in all the systems overlapped very well, indicating that our MD simulations for the four systems had reasonably converged.

### 2.1. AuNPs Prevent the β-Sheet Formation of Aβ Peptides and Prolong the Progress of the Aggregation of Aβ Peptides

**Figure 1:**
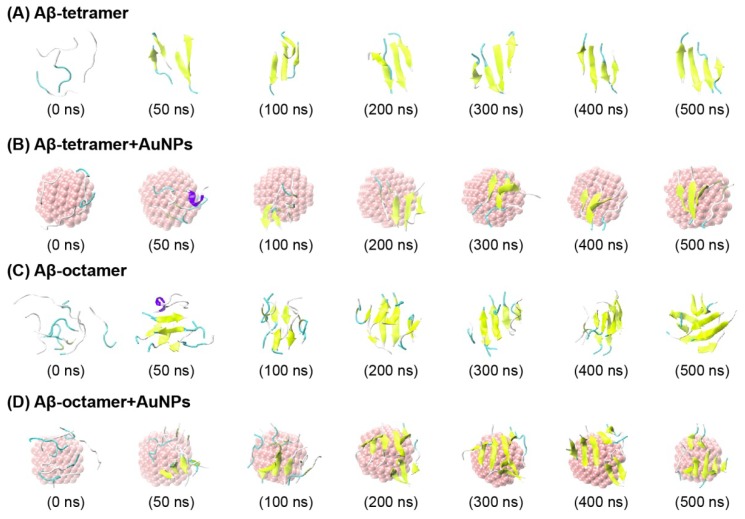
Detailed analysis of a representative molecular dynamics (MD) trajectory starting from the initial state for the Aβ-tetramer system (**A**), Aβ-tetramer + AuNPs system(**B**), Aβ-octamer system (**C**), and Aβ-octamer + AuNPs system (**D**). Snapshots at seven different time points and the top view of the snapshot generated at *t* = 500 ns. The peptides are represented as cartoons, with the β sheet in yellow, the coil in cyan and the other secondary structure in white and purple. The AuNPs are in van der Waals (vdW) representation in pink. For clarity, counter ions and water molecules are not shown.

To characterize the conformation of Aβ peptides in the presence/absence of AuNPs, in Figure 1, we plot the snapshots at seven different time points as the time evolution in the representative MD run for each system. From the snapshots in Figure 1, we can clearly see that in the absence of AuNPs, starting from a random state, in Figure 1A for the Aβ tetramer system, the four peptides adopt different conformations of four-stranded β-sheets from 50 ns. However, in the presence of AuNPs (Figure 1B), all of the Aβ peptides, in the Aβ tetramer + AuNPs system, visit the random coil at 50 ns; as time evolves, the four peptides visit two- and three-stranded β-sheets. These β-sheets form and dissociate during 0–500 ns, indicating that the peptides are not trapped in a single low energy basin during the simulation. On the basis of the snapshot, we hypothesize that the AuNPs inhibit the aggregation of Aβ peptides by prolonging the lag time for Aβ nucleation.

To further probe the effect of AuNPs on the conformation of Aβ peptides, we calculated the secondary structure (α-helix, β-sheet, coil, β-strand, turn and bend) of each trajectory by discarding the first 100 ns of data of all the MD runs for the Aβ tetramer and Aβ tetramer + AuNPs systems (Table 1). In each system, the α-helix, β-strand and bend structures are negligible, with a percentage of <6%. The β-sheet percentage is 40.01% in the isolated Aβ tetramer systems, while it decreased to 27.28% in Aβ tetramer + AuNPs complex systems, much lower than that of isolated Aβ tetramer systems. In contrast, the coil percentages in isolated Aβ tetramer systems are much lower than those in Aβ tetramer + AuNPs complex systems. These secondary structure analysis results also suggest that AuNPs prevents β-sheet structure formation of the Aβ tetramer systems by inducing it into the random-coil conformation.

**Figure 2:**
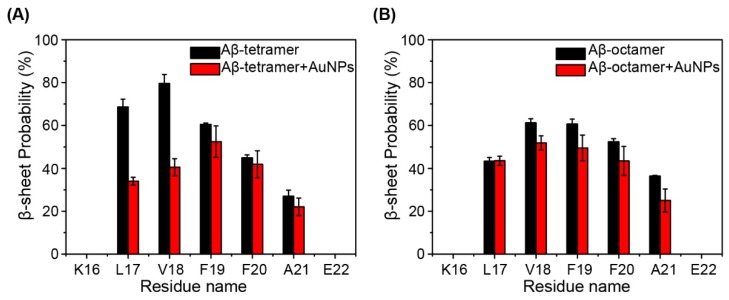
Calculated β-sheet probability of each residue of Aβ (16-22) peptides in (**A**) the Aβ tetramer system (red) and Aβ tetramer + AuNPs system (black) and (**B**) the Aβ octamer system (red) and the Aβ octamer + AuNPs system (black).

After comparing the difference of the secondary structure among the four systems, we further calculated the β-sheet probability of each amino acid residue of Aβ(16-22) peptides, as shown in Figure 2A. We can clearly see that β-sheet probabilities for residues L17-V18 are strongly reduced by AuNPs in the Aβ tetramer + AuNPs complex, compared to those of residues in the isolated Aβ tetramer system. In the Aβ tetramer system, residues L17-V18-F19-F20-A21 in the CHC region have 26.9–79.5% probabilities to adopt β-sheet conformation, with L17 and V18 having a high β-sheet probability of 68.5% and 79.5%. However, in the Aβ tetramer + AuNPs system, this region has a distinctly reduced β-sheet probability of 18.9–49.1%, with a probability of 30.5% for L17 and 36.1% for V18. Taken together, these results demonstrate that the presence of AuNPs significantly prevents β-sheet formation of the Aβ(16-22) tetramer.

To determine whether the effect of AuNPs occurs for different Aβ peptides concentration, we carried out MD simulations of an Aβ(16-22) octamer with/without AuNPs. In Figure 1C for the Aβ octamer system, the eight peptides visit four- and eight-stranded β-sheet alignments from 50 ns. In the Aβ octamer + AuNPs system (Figure 1D), the eight peptides visit three-stranded β-sheet alignments at 50 ns and 100 ns, and after 200 ns, the eight peptides adopt seven- or eight-stranded β-sheet alignments. In Table 1, the β-sheet percentage is 36.29% in the isolated Aβ octamer systems, while it varies from 30.49% in the Aβ octamer + AuNPs complex systems. In Figure 2B, residues L17-V18-F19-F20-A21 in the CHC region have 36.4-61.2% probabilities to adopt the β-sheet conformation in the Aβ octamer system; in the Aβ octamer + AuNPs system, this region has a distinctly reduced β-sheet probability of 25.0-51.9%. In summary, these results demonstrate that the presence of AuNPs also prevents β-sheet formation of the Aβ(16-22) octamer.

It has been found that bare AuNPs inhibit Aβ fibrillation and redirect Aβ, forming fibrils and oligomers [21]. Recently, an experiment reported that the effect of AuNPs on Aβ peptides is size-dependent, and the small AuNPs could significantly postpone the process Aβ fibrillation [16]. Our data agree well with these experiments. From our results, we can conclude that AuNPs with the AuNP: Aβ molar ratio >1:8 can significantly inhibit the β-sheet formation of Aβ(16-22) peptides.

### 2.2. Interactions of AuNPs with Aβ(16-22) Peptides Competes with the Aβ-Aβ Interaction

**Figure 3:**
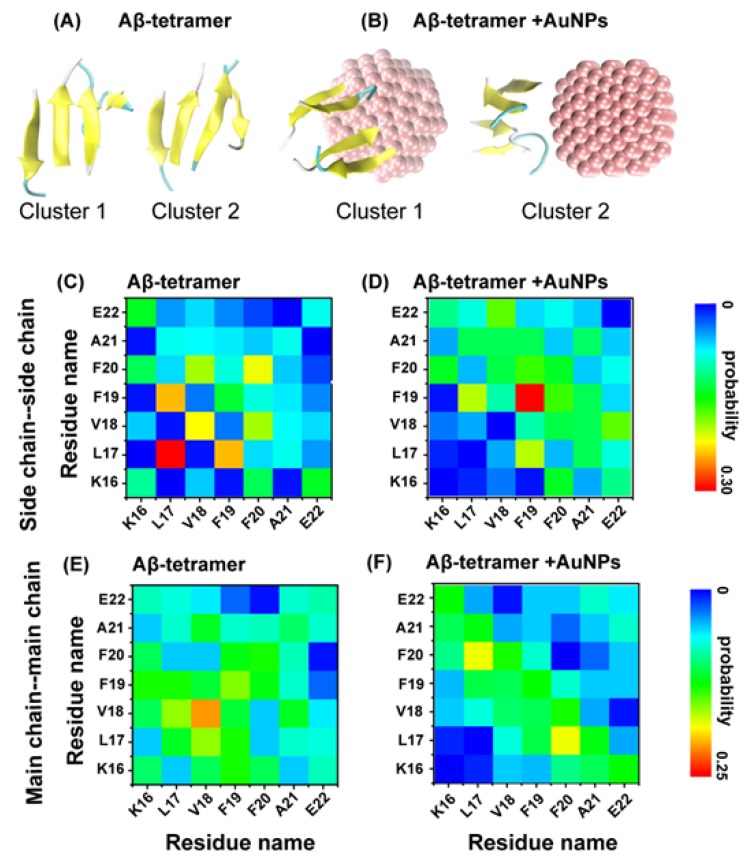
Representative conformations of the top two most-populated clusters for the Aβ tetramer in the absence (**A**) and presence (**B**) of AuNPs. Side-chain-side-chain (SC-SC) and main-chain-main-chain (MC-MC) contact probability maps for Aβ tetramer in the absence (**C**,**E**) and presence (**D**,**F**) of AuNPs. The Aβ tetramer is shown in new cartoon representation. The peptides are colored in yellow. The AuNPs are in vdW representation and colored in pink.

To investigate the influence of AuNPs on the atomic structures of the Aβ tetramer, we performed a chain independent RMSD-based cluster analysis. Using a C_α_–RMSD cutoff of 0.3 nm, the centers of the top two most-populated clusters are shown in Figure 3A,B. The Aβ tetramer populates various parallel and anti-parallel registers in the absence/presence of AuNPs. To better understand the primary peptide-peptide interactions destroyed by AuNPs and the key residues for β-sheet formation, we plot the interpeptide main-chain-main-chain (MC-MC) and side-chain-side-chain (SC-SC) contact probabilities between all pairs of residues for the top two clusters of the Aβ tetramer in the absence and presence of AuNPs in Figure 3.

The residue-residue contact probability maps in these two systems display distinct SC-SC and MC-MC interaction patters, implying that AuNPs significantly impacts the interpeptide interactions. Figure 3C,D shows that the Aβ(16-22) tetramer in the absence of AuNPs is essentially stabilized by SC-SC interactions of L17-L17 (with a contact probability of 33.5%), L17-F19 (24.2%), V18-V18 (22.4%) and F20-F20 (21.6%) and MC-MC interactions of V18-V18 (25%), L17-V18 (19.3%) and F19-F19 (18.6%) pairs. However, in the presence of AuNPs, we find that although the peptides still adopt mainly an antiparallel alignment, the MC-MC contact probabilities are dramatically decreased (for L17-L17 and for L17-F19) in Figure 3E. Significantly reduced SC-SC contact probabilities are also observed in Figure 3F for Aβ(16-22) tetramer + AuNPs. The dramatic decrease of contact probabilities for these hydrophobic/aromatic residue pairs reflects the influence of hydrophobic interactions between AuNPs and the hydrophobic/aromatic residues of Aβ(16-22) peptides. In these two systems, we plot the residue-residue contact probability maps to display the distinct MC-MC and SC-SC interaction patterns, suggesting that AuNPs have a strong impact on the interpeptide interactions. Overall, the presence of AuNPs significantly weakens the interpeptide MC-MC and SC-SC interactions. This is consistent with our previous computational studies that the nanoparticles prevent the formation of amyloid-β by weakening the interpeptide interactions [9,10,11].

**Figure 4:**
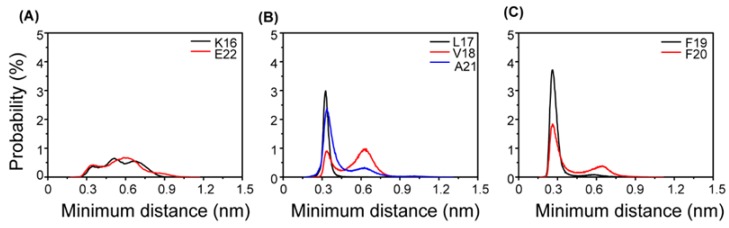
Probability distribution of the minimum distance between the side chain of each residue.and AuNPs’ surface in the Aβ tetramer/octamer + AuNPs system. The distance between AuNPs’ surface and (**A**) K16, E22, (**B**) L17, V18, A21, (**C**)F19, F20.

In order to explore the physical driving forces underlying the β-sheet inhibition and destabilization by AuNPs, the probability distribution of the minimum distance between the side chain of Phe and AuNPs surface are plotted in Figure 4. Two probability peaks are seen for each residue, with a dominant peak centered at 0.35 nm for the hydrophobic (HP) residues L17-V18-A21. The probabilities of these HP residues at 0.35 nm are all greater than those of K16 and E22. In particular, at 0.28 nm, the aromatic residues F19 and F20 have the highest probability. These data indicate that the HP residues L17-A21 have stronger interactions with AuNPs than the hydrophilic residues K16 and E22. Interestingly, in the Aβ tetramer/octamer + AuNPs system, the interaction between the aromatic residue F19 and AuNPs is the strongest. The probability of Phe19 at 0.28 nm is greater than that of Phe20. These data indicate that the interaction between the aromatic residue Phe19 and AuNPs is stronger than that between Phe20 and AuNPs in the Aβ tetramer/octamer + AuNPs system. The results are consistent with our previous data [11], which proposed that the HP residues and aromatic residues play important roles in the peptide-nanoparticles interactions. Experiments and MD simulations also reported that aromatically-rich residues are most frequently mentioned among natural amino acids known as strong Au-binding sites [24,25,26].

**Figure 5:**
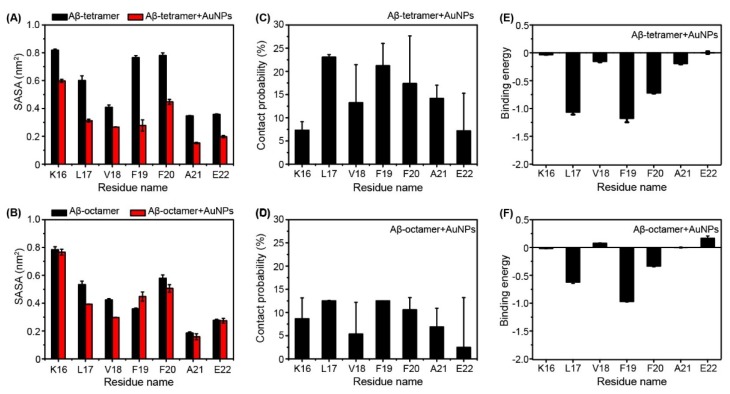
Solvent accessible surface area of each residue as a function of amino acid residue (**A**) for the Aβ tetramer (**B**) and the Aβ octamer in the absence (black) and presence (red) of AuNPs. Residue-based binding interaction analysis: contact probability (**C**,**D**) and binding free energy (in kcal mol^−1^) (**E**,**F**).

It is instructive to look at the role of water in all of the MD simulations. We further calculated the solvent accessible surface area (SASA) of each residue of the Aβ tetramer and Aβ octamer in the absence (black) and presence of AuNPs in Figure 5. In Figure 5A, we can clearly see that the SASA of each residue of Aβ tetramer in the absence of AuNPs is much larger than that in the presence of AuNPs. Especially, the SASA of F19 and F20 are much larger in the absence of AuNPs than in the presence of AuNPs. This indicates that AuNPs have a great effect on residues F19 and F20. Previous experimental [27] and computational studies [28,29] reported that the expulsion of interfacial water molecules is a key event in the aggregation of amyloid peptides such as Aβ(16-22) [27,28].

To further identify the most favorable residues for AuNPs binding, we computed the binding percentage of AuNPs with each amino acid residue using the last 100 ns of data of each MD trajectory. The calculated binding percentage is shown in Figure 5C. It is observed that L17, F19 and F20 have the highest probability/affinity to interact with AuNPs in the Aβ tetramer + AuNPs system. Figure 5D shows that AuNPs have low binding free energy with residues L17, F19 and F20. The binding free energy calculation agreed well with the β-sheet probability of each residue in Figure 2, indicating the important role of AuNPs in preventing the aggregation of the Aβ(16-22) peptide. In the previous studies, it has been proposed that hydrophobic and aromatic stacking interactions play important roles in the formation and stabilization of Aβ(16-22) fibrils [30,31]. The inter-peptide interactions responsible for Aβ(16-22) aggregation would hinder the strong Aβ-AuNPs interactions, therefore inhibiting the nucleation process.

However, in Figure 5B, the SASA of the Aβ octamer has little difference between the Aβ octamer in the presence of AuNPs and in the absence of AuNPs. This indicates that AuNPs are too small to supply enough interaction surface for the Aβ octamer. Interestingly, L17 and F20 also display relatively higher interaction probabilities and lower binding energy in the Aβ octamer + AuNPs system in Figure 5E,F.

From Figure 5, we can see that the two residues (L17 and F20) of the Aβ tetramer/octamer strongly interact with AuNP molecules via hydrophobic interactions. During the progress of the interaction, the adjacent aromatic and hydrophobic residues are protected from being exposed to water, resulting in a much higher β-sheet percentage in this region.

## 3. Materials and Methods

To investigate the effects of AuNPs on Aβ(16-22) peptides, four systems have been studied: Aβ tetramer, Aβ tetramer + AuNPs, Aβ octamer and Aβ octamer + AuNPs. Here, for brevity, we use Aβ for Aβ(16-22). The Aβ(16-22) peptide consists of seven residues (Ace-KLVFFAE-NH2) blocked by acetyl and amine groups as determined experimentally [8]. To mimic the experimental neutral pH condition, the side chain of Lys was protonated (Lys+), and that of Glu was deprotonated (Glu-). Both of the four-peptide chains in the initial state of the Aβ(16-22) tetramer and the eight peptide chains in the initial state of the Aβ(16-22) octamer had random characteristics, similar to those in our previous studies [9,11]. Four systems were placed in a rectangular box of SPC water molecules [32] with a minimum distance to the water box wall of 1.0 nm. The total numbers of atoms for the four systems were 11,000, 15,610, 14,626 and 20,094. The number of atoms for Au nanocluster was 248.

### 3.1. Aβ Tetramer and Aβ Octamer Systems

The starting states of the Aβ tetramer and the Aβ octamer with a random character for each chain are shown in Figure 1A,C.

### 3.2. Aβ Tetramer + AuNPs Complex and Aβ Octamer + AuNPs Complex Systems

The initial states of the Aβ tetramer and Aβ octamer in the complex are the same as in the Aβ tetramer and Aβ octamer system. The Au clusters are constructed as a sphere with diameters of 1 nm. The minimum distance between AuNPs and the Aβ tetramer is 3.3 nm, and the minimum distance between AuNPs and the Aβ octamer is 3.5 nm.

### 3.3. MD Simulation Details

All MD simulations were performed in the isothermal-isobaric (NPT) ensemble. The MD simulations were performed using the GROMACS software package [33]. Following several computational studies of Aβ(16-22) [28,34,35,36], the GROMOS96 43A1 force field [37] was used to describe intramolecular and intermolecular interactions. The temperature was maintained close to 310 K by weak coupling to an external temperature bath [38] with a coupling constant of 0.1 ps, and the pressure as kept at 1 bar using a coupling time of 1.0 ps. Constraints were applied for bond lengths using the SETTLE algorithm [39] for water and LINCS [40] for the peptides and AuNPs. This allowed an integration time step of 2 fs. A twin-range cutoff of 1.0/1.4 nm was applied for van der Waals interactions. A reaction-field correction (with a cutoff of 1.4 nm) with dielectric permittivity ε = 78 was used for the long-range electrostatic interactions. All visualizations were made using the VMD tool [41]. Au atoms of AuNPs are uncharged in accordance with Hummer et al. [42], and the Lennard–Jones parameters for the protein-AuNP and water-AuNP interactions were obtained using the Lorentz–Berthelot rule [43].

### 3.4. Analysis Methods

Analysis was performed using our in-house codes and the GROMACS facilities. We discarded the first 100 ns of each MD in order to remove the bias of the initial states. The structural properties of each system were therefore based on a total of 3.2 μs.

The MD trajectories were analyzed using several parameters. These include the secondary structure content using the DSSP program [44], the percentage of β-sheet and the probability of residue-residue contacts. If the angle of N-H … O was ≥150 and the distance between N and O atoms was ≤3.5 Å degrees, we considered that the hydrogen bond was formed. The probability density function (PDF) of the number of H-bonds and the number of H-bond was analyzed for four systems.

We used the MM-GBSA method [45] implemented in the GROMACS package to calculate the binding energy between the AuNPs and the Aβ peptides. In the MM/GBSA method, the binding free energy (∆G_binding_) between a ligand and a receptor was ∆G_binding_ = ∆E_MM_ + ∆G_solv_ − T∆S. In the formula, ∆E_MM_ is the gas phase energy, consisting of electrostatic (∆E_elec_) and van der Waals (∆E_vdW_) terms. ∆G_solv_ is the sum of polar solvation energy (∆G_polar_) and nonpolar solvation component ∆G_surf_. ∆G_polar_ is calculated by the GB model [46], and ∆G_surf_ is estimated by the solvent accessible surface area (SASA). As the binding free energy (∆G_binding_) reported here was the relative binding free energy, the contribution of conformational entropy of peptides was ignored in accordance with a number of previous computational studies [9,47,48,49,50]. Therefore, the binding free energy was calculated using ∆G_binding_ = ∆E_MM_ + ∆G_solv_ in this work.

## 4. Conclusions

We studied the effect of AuNPs on the secondary structure of the Aβ(16-22) peptides by performing eight 500 ns molecular dynamics simulations starting from random states. Our MD simulations demonstrate that AuNPs of 1 nm in diameter can greatly prevent β-sheet formation. From the analysis of our simulation, we can conclude that the inhibition depends on the concentration of Aβ(16-22) peptides. Interestingly, we also find that the inhibition of β-sheet formation by AuNPs results from the same physical forces: hydrophobic interactions. Overall, our computational study provides evidence that AuNPs are likely to inhibit the aggregation of Aβ(16-22) peptides. The inhibition depends on the concentration of Aβ(16-22). Thus, this study displayed a full picture that AuNPs can inhibit Aβ(16-22) aggregation, indicating that inorganic nanoparticles might be used as drug candidates for treatment of AD.

## Figures and Tables

**Table 1:** Average secondary structure propensity of the Aβ(16-22) peptides with and without AuNPs.

Reasearch System	α-Helix	β-Sheet	β-Strand	Turn	Random Coil	Bend
Abeta tetramer	<0.01 ± 0.730	40.01 ± 1.837	2.61 ± 1.094	1.52 ± 0.068	53.12 ± 0.078	2.35 ± 1.094
Abeta tetramer + AuNPs	<0.01 ± 0.139	27.28 ± 3.360	5.11 ± 0.087	1.48 ± 0.348	60.95 ± 2.785	5.19 ± 0.087
Abeta octamer	<0.01 ± 0.354	36.29 ± 0.142	4.18 ± 0.794	1.70 ± 1.580	53.65 ± 0.303	3.33 ± 0.794
Abeta octamer + AuNPs	<0.01 ± 0.001	30.49 ± 3.367	4.79 ± 0.811	0.28 ± 0.098	59.66 ± 2.656	3.85 ± 0.811

## Supplementary Materials

Supplementary materials can be found at http://www.mdpi.com/1422-0067/19/6/1815/s1.
