# Supplementary material for: Exploring the Mechanism of Inhibition of Au Nanoparticles on the Aggregation of Amyloid-β(16-22) Peptides at the Atom Level by All-Atom Molecular Dynamics
Source: Int J Mol Sci. 2018 Jun 20;19(6):1815. doi: 10.3390/ijms19061815 (PMC6032210; doi:10.3390/ijms19061815)
Supplement: Supplementary file 1 [file ijms-19-01815-s001.pdf]

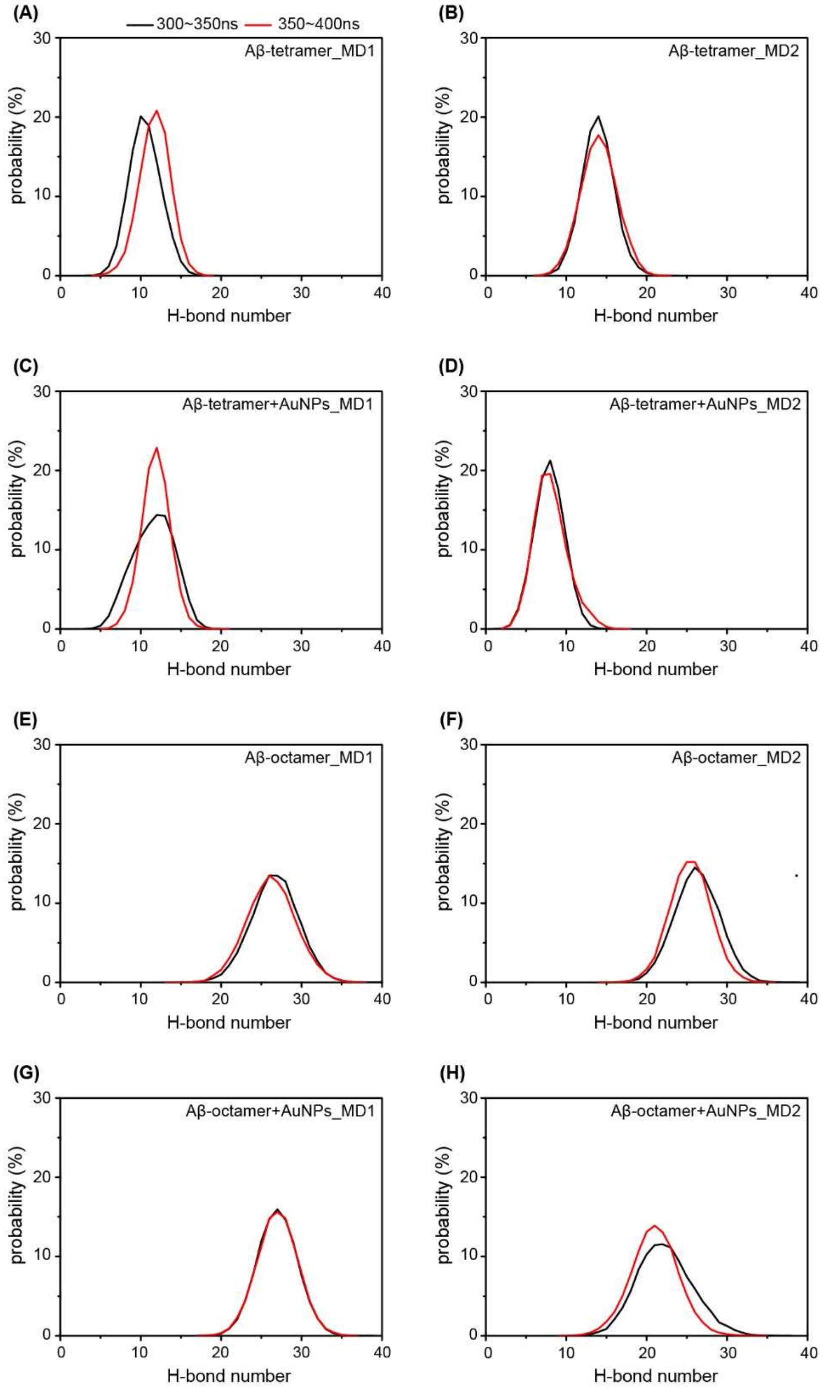

Figure S1: Convergence check of the REMD runs of Aβ(16-22). Probability distribution of end-to-end distance (the Ca-Ca distance between K16 and E22) of Aβ (16-22). (A) at neutral pH; (B) at acidic pH.
